# Pathways to Future Depression Among University Students: The Role of Interpersonal Needs, Emotion Regulation and Meaning in Life

**DOI:** 10.1002/mpr.70084

**Published:** 2026-06-15

**Authors:** José Enrique Layrón, José H. Marco, Rafael Salom Borrás, Sandra Pérez Rodríguez

**Affiliations:** ^1^ Universidad Internacional de Valencia (VIU) Valencia Spain; ^2^ Universidad Europea de Valencia (UEV) Valencia Spain; ^3^ Department of Personality Assessment and Psychological Treatments. Universidad de Valencia Valencia Spain; ^4^ Clínica Nuestra Señora de la Paz Orden Hospitalaria San Juan de Dios Madrid Spain

**Keywords:** depression, emotion regulation, interpersonal needs, meaning in life, university students

## Abstract

**Objectives:**

This study examined prospective pathways to future depressive symptoms among university students, focusing on interpersonal needs, emotion regulation strategies, meaning in life, and suicide risk markers.

**Methods:**

A longitudinal design was conducted with 737 Spanish university students assessed at baseline (T1) and 225 reassessed 14 weeks later (T2), reflecting substantial attrition across follow‐up. Measures included depressive symptoms, thwarted belongingness, perceived burdensomeness, emotion regulation strategies (cognitive reappraisal and expressive suppression), meaning in life, non‐suicidal self‐injury (NSSI) frequency, and suicidal ideation frequency. Structural equation modeling in JASP (lavaan backend) tested a theory‐driven model estimating direct and indirect effects on depressive symptoms at follow‐up, controlling for baseline depression. Sensitivity analyses were performed excluding baseline depression.

**Results:**

Baseline depressive symptoms were the strongest predictor of future depression. Thwarted belongingness predicted higher expressive suppression and lower meaning in life, whereas perceived burdensomeness showed a direct association with depressive symptoms at follow‐up. When baseline depression was excluded, thwarted belongingness indirectly predicted future depressive symptoms through expressive suppression. The final models explained up to 42% of the variance in depressive symptoms at T2.

**Conclusions:**

Interpersonal difficulties and maladaptive emotion regulation processes predicted the persistence of depressive symptoms among university students. Interventions targeting social connectedness and expressive suppression may help reduce the risk of sustained depressive symptomatology in this population.

## 1 Introduction

Depressive symptoms are highly prevalent among university students and often exceed rates observed in the general population (Lei et al. 2016; Rotenstein et al. 2016). In Spain, 18.9% of university students reported a major depressive episode in the past year, and lifetime prevalence reached 23.1% (Ballester et al. 2020).

Within this population, interpersonal variables may play an important role in the development and maintenance of depressive symptoms. In particular, the Interpersonal Theory of Suicide highlights perceived burdensomeness and thwarted belongingness as key constructs associated with suicidal ideation and psychological distress (Joiner 2005; Van Orden et al. 2012). In university samples, these interpersonal needs have been linked not only to suicidal ideation, but also to depressive symptoms, with depression often occupying a central role in this network of associations (Han and Lee 2021; Poindexter et al. 2020). Depressive symptoms have also been related to suicide risk markers such as non‐suicidal self‐injury (NSSI) in young adults (Zhou et al. 2023).

In addition to interpersonal risk factors, emotion regulation strategies and meaning in life may function as relevant protective resources in university populations. Cognitive reappraisal has been associated with better psychological adjustment and lower depressive symptoms, whereas expressive suppression is often linked to greater distress and poorer social functioning (Y. Liu et al. 2019; Sánchez‐López and Reivan‐Ortiz 2024). Longitudinal evidence further suggests that expressive suppression predicts subsequent reductions in social support and relationship quality, supporting its potential role in prospective links between interpersonal difficulties and depressive symptoms (Srivastava et al. 2009). By contrast, cognitive reappraisal enables a more flexible reinterpretation of stressful experiences and has been associated with lower depressive symptoms among university students (Álvarez‐Huerta et al. 2023; Y. Liu et al. 2019).

Meaning in life may also act as an important protective factor. Conceptualized as a multidimensional construct encompassing coherence, purpose, and existential significance (Martela and Steger 2016), meaning in life has been linked to greater well‐being and lower emotional distress. In university students, it has been described as a buffer against depressive symptoms and suicidal ideation, suggesting that it may help reduce vulnerability in contexts of distress (Baquero‐Tomás et al. 2023; Layrón‐Folgado et al. 2022).

Although previous literature has explored the relationships between suicidal ideation and variables such as meaning in life, cognitive reappraisal, expressive suppression, thwarted belongingness, perceived burdensomeness, depression, and NSSI in university students (Layrón‐Folgado et al. 2022; Ogbonnaya et al. 2024), to the best of our knowledge no studies in Spanish university samples have simultaneously examined these constructs within a theory‐driven, multivariate prospective model.

Accordingly, the present study tests a more comprehensive framework in which interpersonal needs (thwarted belongingness and perceived burdensomeness) are hypothesized to predict future depressive symptomatology both directly and indirectly through protective and regulatory processes—namely meaning in life, cognitive reappraisal, and expressive suppression—as well as through suicide risk markers (NSSI frequency and suicidal ideation frequency), while controlling for baseline depression. Although previous work suggests that expressive suppression may mediate the association between interpersonal difficulties and distress (Srivastava et al. 2009), the joint and unique contributions of these risk and protective factors within a single prospective model remain unclear in Spanish university populations.

From a theoretical perspective, interpersonal needs may function as distal vulnerability factors for later depressive symptoms in university students. Specifically, thwarted belongingness and perceived burdensomeness may contribute to psychological distress by weakening perceived social connectedness and reinforcing negative self‐evaluative processes (Joiner 2005; Van Orden et al. 2012). In turn, these interpersonal difficulties may be associated with less adaptive emotion regulation, particularly greater expressive suppression, as well as with lower meaning in life, a psychological resource consistently linked to better adjustment and lower emotional distress (Baquero‐Tomás et al. 2023; Layrón‐Folgado et al. 2022; Y. Liu et al. 2019; Srivastava et al. 2009). At the same time, suicide‐related risk markers such as suicidal ideation and NSSI may reflect increased vulnerability in the context of interpersonal and emotional distress, given their documented associations with depressive symptomatology in young adults and university students (Whitlock et al. 2013; Zhou et al. 2023). Taken together, these relationships suggest that interpersonal difficulties may contribute to future depressive symptoms both directly and indirectly through emotion regulation, meaning in life, and suicide‐related vulnerability.

Therefore, the main objectives of the present study were: (1) to analyze the relationships among interpersonal needs (thwarted belongingness and perceived burdensomeness), protective and regulatory processes (meaning in life, cognitive reappraisal, and expressive suppression), NSSI frequency, suicidal ideation frequency, and depressive symptoms at baseline and follow‐up; (2) to test a theory‐driven structural equation model estimating the unique prospective and indirect effects of thwarted belongingness and perceived burdensomeness on follow‐up depression through meaning in life, cognitive reappraisal, expressive suppression, NSSI frequency, and suicidal ideation frequency, while controlling for baseline depression; and (3) as a sensitivity analysis, to estimate a parsimonious structural equation model excluding baseline depressive symptomatology in order to compare direct and indirect pathways from interpersonal needs to follow‐up depressive symptoms using standardized coefficients.

## 2 Method

### 2.1 Procedure

For participant recruitment, the researchers contacted the deans of several university faculties in Valencia, Spain. Ultimately, six faculties agreed to participate in the study: Psychology, Occupational Therapy, Nutrition, Podiatry, Nursing, and Speech Therapy. Students were informed about the study through in‐class announcements and emails sent by academic coordinators. Those who chose to participate signed an informed consent form in the classroom prior to the assessment. Participation was entirely voluntary and anonymous. As incentives, academic credits and a certificate of participation were offered.

Eligibility criteria included being of legal age, actively enrolled in a university program at the time of the study, and possessing a smartphone. Students who did not meet these criteria or failed to sign the informed consent form were excluded. No exclusion criteria were applied regarding prior history of suicidal behavior. The study was approved by the university's ethics committee.

After providing informed consent, participants completed the baseline assessment (T1) using the MEmind smartphone application, which enables longitudinal administration of questionnaires and linkage of responses across time points (Barrigón et al. 2017). This study is part of a broader ecological momentary assessment (EMA) project in which participants were assessed daily over a 3‐month period using the same application. Participants were invited to complete repeated daily assessments during this period. Follow‐up assessment (T2) data used in the present study correspond to those participants who completed the EMA protocol and responded to the post‐assessment after this period. Follow‐up assessment (T2) was conducted 14 weeks later. Data were collected between September 2018 and January 2020, and no personally identifying information was collected.

As part of the study's safety protocol, participants reporting high levels of suicidal ideation through the app received an automated message informing them that they could contact the research team by email to access free psychological support. When no help was requested, follow‐up reminders were sent periodically, also asking whether the participant was already receiving psychological or professional support. Given the anonymous nature of the study, support was offered through participant‐initiated contact rather than direct individualized outreach.

### 2.2 Participants

A total of 975 university students who met the inclusion criteria were approached in their classrooms and initially expressed willingness to participate. Of these, 737 completed all questionnaires at the baseline assessment (T1) using the *MEmind* app. From this group, 225 students completed the entire follow‐up assessment at T2 (14 weeks later).

Participants ranged in age from 18 to 40 years (*M* = 22.51; SD = 4.06), with 95% being under the age of 26. Regarding gender, 165 participants (73.3%) were female, and 60 (26.7%) were male. Most of the sample reported being single (*n* = 196; 86.7%), while 29 participants (13.3%) were married or cohabiting with a partner. In terms of educational background, 13 participants (5.8%) had already obtained a university degree, while 212 (94.2%) had completed high school.

### 2.3 Instruments

*The Emotion Regulation Questionnaire* (ERQ; Gross and John 2003; Spanish version: Cabello et al. 2013). This instrument assesses two emotion regulation strategies and consists of 10 items grouped into two subscales: (1) Cognitive Reappraisal (6 items) and (2) Expressive Suppression (4 items). The instrument has demonstrated acceptable reliability (*α* = 0.75 for cognitive reappraisal; *α* = 0.79 for expressive suppression). In the current student sample, internal consistency was also adequate (*α* = 0.78 for both subscales, respectively). An example item for cognitive reappraisal is: *“When I want to feel less negative emotion, I change the way I'm thinking about the situation,” and for expressive suppression: “I control my emotions by not expressing them”.*

*The Interpersonal Needs Questionnaire* (INQ‐15; Van Orden et al. 2012; Spanish version: Silva et al. 2018). This 15‐item instrument assesses two subscales: (1) Thwarted Belongingness and (2) Perceived Burdensomeness. The Spanish version has demonstrated adequate reliability, with Cronbach's alpha coefficients of *α* = 0.96 for perceived burdensomeness and *α* = 0.78 for thwarted belongingness. In the current study, internal consistency was satisfactory (*α* = 0.82 for perceived burdensomeness; *α* = 0.80 for thwarted belongingness). A sample item for thwarted belongingness is: *“These days, I feel disconnected from other people,” and for perceived burdensomeness: “These days, I think I am a burden on society”.*

*The Inventory of Statements About Self‐Injury* (ISAS; Klonsky and Glenn 2009; Spanish version: Castro‐Silva et al. 2016). This questionnaire evaluates both the presence and frequency of non‐suicidal self‐injury (Part 1), as well as its functions (Part 2). Part 1 assesses the lifetime frequency of 12 types of self‐injury, while Part 2 explores 11 functions grouped into intrapersonal and interpersonal domains (e.g., emotion regulation, suicide avoidance, establishing boundaries or interpersonal connection). The original version showed good internal consistency (*α* = 0.88 and 0.80, respectively). In the present study, only Part 1 was used, assessing the frequency of NSSI at T1.

*The Patient Health Questionnaire* (PHQ‐9; Kroenke et al. 2001; Spanish version: Diez‐Quevedo et al. 2001). This instrument is used both for the potential diagnosis of depression based on DSM criteria and to assess symptom severity. It consists of nine items measuring the presence of depressive symptoms over the previous 2 weeks. An example item is: *“Little interest or pleasure in doing things.”* The Spanish version has demonstrated reliability similar to the original, and internal consistency in the current sample was acceptable (*α* = 0.72). This measure was used to compare university students according to their current levels of depressive symptoms. PHQ‐9 scores are interpreted as follows: 0–4 = minimal or no symptoms; 5–9 = mild depression; 10–14 = moderate depression; 15–19 = moderately severe depression; 20–27 = severe depression.

*Purpose in Life‐10 (*PIL‐10; Crumbaugh and Maholick 1969; Spanish version: García‐Alandete et al. 2013). We used the abbreviated version of the PIL, composed of 10 items that assess various dimensions of meaning in life. Scores range from 10 to 70, with higher scores indicating greater perceived meaning. An example item is: *“In life I have… No goals or aspirations” to “many goals and defined aspirations.”* This brief version has excellent psychometric properties (*α* = 0.92). In the present study, internal consistency was satisfactory (*α* = 0.88).

*Ad‐hoc items.* Suicidal ideation was assessed using two custom questions: *“During the past week, have you thought about ending your life? How many times?”* Response options were: 0, 1–4 times, 5–50 times, more than 50 times. These brief direct questions were included to reduce participant burden and facilitate feasibility within the broader assessment protocol. Prior literature suggests that brief suicide‐related questions may be useful for initial screening, although the use of validated instruments remains preferable in terms of psychometric robustness and comparability across studies (Horowitz et al. 2012; Millner et al. 2015). In addition, previous research has identified recent suicidal ideation as one of the strongest predictors of future suicide attempts (e.g., Miranda‐Mendizábal et al. 2019).

### 2.4 Statistical Analyses

Statistical analyses were conducted using IBM SPSS Statistics (version 29.0.2) for descriptive statistics and bivariate correlations, and JASP (version 18.3; *lavaan* backend) for structural equation modeling (SEM). Descriptive statistics and Pearson correlations were computed for all variables and depressive symptoms at follow‐up (T2). To assess potential attrition bias, baseline differences between participants who completed the follow‐up assessment (T2) and those who did not were examined using ANOVA for continuous variables, and effect sizes (η^2^) were calculated to estimate the magnitude of differences.

SEM was used to examine prospective associations between interpersonal needs (thwarted belongingness, perceived burdensomeness), emotion regulation (cognitive reappraisal, expressive suppression), meaning in life, suicide‐related variables (NSSI and suicidal ideation frequency), and depressive symptoms at T2, controlling for baseline depressive symptoms. Although NSSI frequency showed a marked floor effect in this community sample, it was retained in the analyses on theoretical grounds, given previous evidence identifying NSSI as a clinically relevant suicide‐related vulnerability marker associated with suicidal ideation, suicide attempts, and elevated suicide risk in young adults and university students (Pérez et al. 2019).

Models were estimated using robust maximum likelihood (MLR; *lavaan* implementation with robust standard errors and Satorra–Bentler scaled χ^2^). Missing data were handled using full information maximum likelihood (FIML). Residual covariances were specified a priori between conceptually related variables. Model fit was evaluated using χ^2^SB, CFI, TLI, RMSEA (90% CI), and SRMR.

Indirect effects were computed as products of path coefficients and tested using robust standard errors (delta method). Model refinement followed a theory‐driven approach: initial models included all hypothesized paths, and non‐significant paths were trimmed using Wald tests when their removal did not compromise model fit or theoretical interpretability. Both full and parsimonious models are reported for transparency. An additional model excluding baseline depressive symptoms was estimated to assess robustness.

## 3 Results

### 3.1 Descriptive Statistics

Table 1 summarizes the descriptive statistics of the study variables. At baseline, participants reported a mean PHQ‐9 total score of 6.06 (SD = 4.15; *n* = 674), indicating, on average, mild depressive symptomatology. In addition to the categorical PHQ‐9 severity breakdown, the sample showed mean scores of 6.63 (SD = 1.50; *n* = 648) for perceived burdensomeness, 11.84 (SD = 3.00; *n* = 651) for thwarted belongingness, 27.57 (SD = 6.78; *n* = 676) for cognitive reappraisal, 13.47 (SD = 5.48; *n* = 665) for expressive suppression, and 57.25 (SD = 8.87; *n* = 676) for meaning in life. Regarding suicide‐related variables, the mean EMA suicidal ideation frequency score was 0.019 (SD = 0.516; *n* = 737), and the mean NSSI frequency score was 0.02 (SD = 0.23; *n* = 737), indicating highly skewed distributions with low overall frequency. Categorically, 98.8% of participants reported no NSSI in the last week, and PHQ‐9 severity categories showed that 84.1% fell within the none/mild range, 10.5% in the moderate range, 4.0% in the moderately severe range, and 1.3% in the severe range.

**TABLE 1:** Statistical descriptives of clinical variables.

Continuous variables			
Variable	*n*	*M*	SD
PHQ‐9 total score	674	6.06	4.15
Perceived burdensomeness	648	6.63	1.50
Thwarted belongingness	651	11.84	3.00
Cognitive reappraisal	676	27.57	6.78
Expressive suppression	665	13.47	5.48
Meaning in life	676	57.25	8.87
EMA suicidal ideation frequency	737	0.019	0.516
NSSI frequency	737	0.02	0.23

Categorical variables			
Variable	Category	*n*	%
NSSI last week (*N* = 737)	No	728	98.8
	Yes	9	1.2
Frequency of NSSI	Never	727	98.6
	Once	6	0.8
	2–4 times	2	0.3
	5–10 times	1	0.1
	More than 10 times	1	0.1
Depressive symptoms (*N* = 674)	None or mild depression	567	84.1
	Moderate depression	71	10.5
	Moderately severe depression	27	4.0
	Severe depression	9	1.3

*Note:* Continuous variables are presented as *n*, mean, and standard deviation; categorical variables are presented as frequency and percentage. Sample sizes vary due to missing data.

Abbreviations: EMA = ecological momentary assessment; NSSI = non‐suicidal self‐injury; PHQ‐9 = Patient Health Questionnaire‐9.

### 3.2 Attrition Analyses

To examine potential attrition bias, baseline differences between participants who completed the follow‐up assessment (T2; *n* = 225) and those who did not (*n* = 512) were analyzed. In this regard, participants who completed the follow‐up reported slightly lower levels of perceived burdensomeness (*F* = 4.19, *p* = 0.041, *η* ^2^ = 0.006) and thwarted belongingness (*F* = 12.10, *p* < 0.001, *η* ^2^ = 0.018), and higher levels of cognitive reappraisal (*F* = 9.59, *p* = 0.002, *η* ^2^ = 0.014) and meaning in life (*F* = 8.20, *p* = 0.004, *η* ^2^ = 0.012) at baseline. They also showed lower depressive symptoms compared to those who did not complete T2 (*F* = 2.51, *p* = 0.113, *η* ^2^ = 0.004), although this difference did not reach statistical significance.

However, although several of these differences reached statistical significance, effect sizes were small (*η* ^2^ ranging from 0.002 to 0.018). No significant differences were observed in expressive suppression (*F* = 1.46, *p* = 0.227, *η* ^2^ = 0.002), suicidal ideation frequency (*F* = 2.28, *p* = 0.132, *η* ^2^ = 0.003), or NSSI frequency (*F* = 0.17, *p* = 0.678, *η* ^2^ = 0.000).

### 3.3 Correlation Analyses

Pearson correlations were computed using pairwise available data; thus, the sample size varied across correlation pairs depending on missingness. For correlations involving depressive symptoms at T2, pairwise Ns ranged approximately from 204 to 225. In relation to the first objective, correlation analyses showed that future depressive symptomatology was positively and statistically significantly associated with baseline depressive symptomatology (*r* = 0.645, *p* = 0.000), thwarted belongingness (*r* = 0.316, *p* = 0.000), perceived burdensomeness (*r* = 0.454, *p* = 0.000), suicidal ideation (*r* = 0.182, *p* = 0.006), expressive suppression (*r* = 0.297, *p* = 0.000), and frequency of NSSI (*r* = 0.258, *p* = 0.031). A negative and statistically significant correlation was also observed between future depressive symptomatology and meaning in life (*r* = −0.384, *p* = 0.000), and cognitive reappraisal (*r* = −0.150, *p* = 0.031) (Table 2).

**TABLE 2:** Correlation analysis of key variables: DS T1 (Depressive Symptoms Pre), DS T2 (Depressive Symptoms Post), emotion regulation strategies, meaning in life, interpersonal needs, frequency and functions of non‐suicidal self‐injury (NSSI), and frequency of suicidal ideation.

	Variable	1	2	3	4	5	6	7	10	11
1	DS T2	—								
2	DS T1	0.645***	—							
3	Cognitive reappraisal	−0.150*	−0.171***	—						
4	Expressive suppression	0.297***	0.311***	−0.086*	—					
5	Meaning in life	−0.384***	−0.478***	0.331***	−0.291***	—				
6	Perceived burdensomeness	0.454***	0.521***	−0.140**	0.253***	−0.452***	—			
7	Thwarted belongingness	0.316***	0.428***	−0.225***	0.347***	−0.560***	0.505***	—		
8	Frequency of suicidal ideation	0.182**	0.083*	−0.049	0.060	−0.114**	0.088*	0.068	—	
9	Frequency of NSSI	0.258***	0.164***	−0.002	0.040	−0.102**	0.195***	0.185***	0.157***	—

*Note:* *** Significant correlation at 0.001 level; ** Significant correlation at 0.01 level; * Significant correlation at 0.05 level.

### 3.4 Structural Equation Model

SEM analyses were estimated in the follow‐up subsample with available T2 data (*n* = 225), using FIML to retain all available information within this subsample. Relative to objective 2, we conducted SEM in JASP (lavaan) with the robust MLR/Satorra–Bentler correction. The hypothesized model showed good fit—SB χ^2^(8) = 7.85, *p* = 0.448; CFI = 0.982; TLI = 0.921; RMSEA = 0.069 (90% CI [0.00, 0.126]); SRMR = 0.033—and explained 41.8% of depressive symptoms at T2. Baseline depressive symptoms strongly predicted later depression (*β* = 0.53, *p* < 0.001). Among interpersonal variables, thwarted belongingness predicted greater expressive suppression (*β* = 0.28, *p* < 0.001) and lower meaning in life (*β* = −0.41, *p* < 0.001), whereas perceived burdensomeness showed no significant associations with the mediators (reappraisal: *β* = −0.00, *p* = 0.986; suppression: *β* = 0.02, *p* = 0.843; meaning in life: *β* = −0.07, *p* = 0.418; NSSI: *β* = 0.22, *p* = 0.211; suicidal‐ideation frequency: *β* = 0.15, *p* = 0.359). None of the mediators showed a significant direct effect on T2 depression when modeled simultaneously (all *ps* ≥ 0.200), and interpersonal predictors did not relate to reappraisal or to NSSI/suicide ideation frequency. Although baseline depressive symptoms emerged as the strongest predictor of follow‐up depression, interpersonal variables remained associated with key intermediate processes, particularly expressive suppression and meaning in life in the case of thwarted belongingness. The only indirect effect approaching significance was for thwarted belongingness via protective factors (*β* = 0.07, *p* = 0.069). All remaining indirect effects were nonsignificant.

**FIGURE 1:**
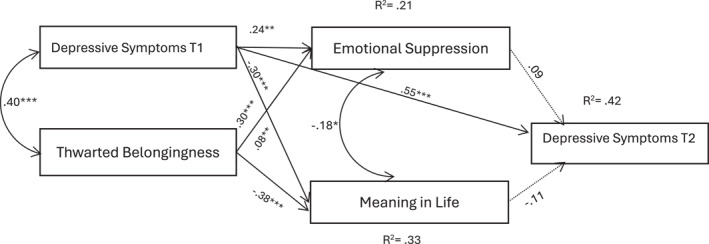
Final structural equation model of depressive symptoms at T2: standardized path coefficients (*β*) and *R* ^2^. *p* < 0.05 (*)*, p* < 0.01 (**), *p* < 0.001 (***).

To improve model parsimony and interpretability, non‐significant paths were removed based on Wald tests, provided that their exclusion did not compromise model fit or theoretical coherence. The parsimonious model fit excellently—SB χ^2^(1) = 0.087, *p* = 0.768; CFI = 1.000; TLI = 1.036; RMSEA = 0.000 (90% CI [0.000, 0.152], *p*‐close = 0.735); SRMR = 0.005—and explained 33% of meaning in life, 20.7% of expressive suppression, and 42.1% of depressive symptoms at T2 (Figure 1). Thwarted belongingness related to lower meaning in life (*β* = −0.384, *p* < 0.001) and higher expressive suppression (*β* = 0.305, *p* < 0.001). Baseline depression related to lower meaning in life (*β* = −0.301, *p* < 0.001) and higher expressive suppression (*β* = 0.238, *p* = 0.006). T2 depression was strongly predicted by baseline depression (*β* = 0.554, *p* < 0.001). Direct paths from meaning in life (*β* = −0.107, *p* = 0.140) and expressive suppression (*β* = 0.087, *p* = 0.219) to T2 depression were not significant. Residual covariances (reported in Table 3) indicated positive overlap between thwarted belongingness and baseline depression (*r* = 0.398, *p* < 0.001) and a negative association between meaning in life and expressive suppression (*r* = −0.180, *p* = 0.035).

**TABLE 3:** Final SEM: Fit indices, structural paths, explained variance, and residual covariances.

	Path/Pair	B	Std *β*	SE	z	*p*
	Thwarted belongingness → expressive suppression	0.875	0.408	0.136	6.444	< 0.001
	Burdensomeness → meaning in life	−0.854	−0.147	0.472	−1.807	0.071
	Thwarted belongingness → meaning in life	−1.352	−0.471	0.262	−5.168	< 0.001
	Burdensomeness → depressive symptoms (T2)	1.140	0.352	0.396	2.879	0.004
	Thwarted belongingness → depressive symptoms (T2)	0.350	0.219	0.157	2.225	0.026
Covariance (exogenous)	Burdensomeness ↔ thwarted belongingness	1.238	0.359	0.322	3.841	< 0.001
Residual covariance	Meaning in life ↔ expressive suppression	−6.719	−0.203	2.637	−2.548	0.011
	Expressive suppression ↔ depressive symptoms (T2)	3.580	0.186	1.461	2.451	0.014
	Meaning in life ↔ depressive symptoms (T2)	−4.988	−0.211	2.540	−1.964	0.050
R^2^	Meaning in life	0.293				
	Expressive suppression	0.166				
	Depressive symptoms (T2)	0.228				

*Note:* Unstandardized coefficients (B) are reported with robust standard errors (SE), z statistics, and *p* values based on the Satorra–Bentler scaled test statistic. Std *β* = standardized coefficient; R^2^ = explained variance. T2 = follow‐up.

To test objective 3, an exploratory model excluding baseline depression fit well—SB χ^2^(6) = 5.19, *p* = 0.520; CFI = 0.988; TLI = 0.946; RMSEA = 0.053 (90% CI [0.00, 0.120]); SRMR = 0.034—and explained 31% of depressive symptoms at T2 (*R* ^2^ = 0.310). Explained variance for mediators was: meaning in life = 0.333, expressive suppression = 0.174, suicidal ideation = 0.053, NSSI = 0.113, and cognitive reappraisal = 0.024. Perceived burdensomeness was positively associated with depressive symptoms at T2 (*β* = 0.309, *p* < 0.001), whereas thwarted belongingness was not (*β* = 0.118, *p* = 0.241). Direct mediator → depression paths were nonsignificant (trend for expressive suppression: *β* = 0.135, *p* = 0.065). Thwarted belongingness showed a significant indirect effect via protective processes (*β* = 0.114, *p* = 0.027) and a significant total indirect effect (*β* = 0.117, *p* = 0.020); indirect effects of burdensomeness were nonsignificant.

**FIGURE 2:**
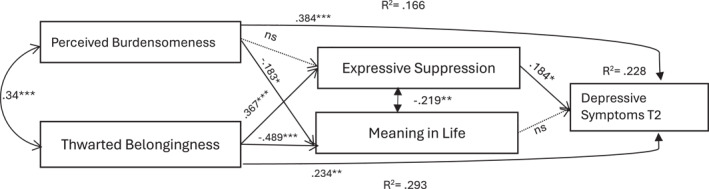
Final structural equation model of depressive symptoms at T2 eliminating depressive symptoms at T1: standardized path coefficients (*β*) and *R* ^2^. *p* < 0.05 (*)*, p* < 0.01 (**), *p* < 0.001 (***).

To improve model parsimony and interpretability, non‐significant paths were removed based on Wald tests, provided that their exclusion did not compromise model fit or theoretical coherence. The final model retained thwarted belongingness and perceived burdensomeness as exogenous predictors of meaning in life and expressive suppression, along with their direct effects on T2 depression, and allowed covariance between exogenous variables. The parsimonious model fit well—SB χ^2^(1) = 0.940, *p* = 0.332; CFI = 0.993; TLI = 0.934; RMSEA = 0.081 (90% CI [0.000, 0.233]); SRMR = 0.027—and explained 29.3% of meaning in life, 16.6% of expressive suppression, and 22.8% of depressive symptoms at T2. Thwarted belongingness related to lower meaning in life (*β* = −0.471, *p* < 0.001) and higher expressive suppression (*β* = 0.408, *p* < 0.001). Depressive symptoms at T2 were predicted by perceived burdensomeness (*β* = 0.352, *p* = 0.004) and by thwarted belongingness (*β* = 0.219, *p* = 0.026). The path from perceived burdensomeness to meaning in life was small and not significant (*β* = −0.147, *p* = 0.071). Residual covariances among endogenous variables were estimated but are omitted from the figure for clarity (see Table 4); the exogenous covariance between burdensomeness and thwarted belongingness was retained (Figure 2).

**TABLE 4:** Final SEM without baseline depression: Structural paths, residual covariances and explained variance.

	*Path/Pair*	*B*	*Std β*	SE	*z*	*p*
	Thwarted belongingness → expressive suppression	0.875	0.408	0.136	6.444	< 0.001
	Burdensomeness → meaning in life	−0.854	−0.147	0.472	−1.807	0.071
	Thwarted belongingness → meaning in life	−1.352	−0.471	0.262	−5.168	< 0.001
	Burdensomeness → depressive symptoms (T2)	1.140	0.352	0.396	2.879	0.004
	Thwarted belongingness → depressive symptoms (T2)	0.350	0.219	0.157	2.225	0.026
Covariance (exogenous)	Burdensomeness ↔ thwarted belongingness	1.238	0.359	0.322	3.841	< 0.001
Residual covariance	Meaning in life ↔ expressive suppression	−6.719	−0.203	2.637	−2.548	0.011
	Expressive suppression ↔ depressive symptoms (T2)	3.580	0.186	1.461	2.451	0.014
	Meaning in life ↔ depressive symptoms (T2)	−4.988	−0.211	2.540	−1.964	0.050
R^2^	Meaning in life	0.293				
	Expressive suppression	0.166				
	Depressive symptoms (T2)	0.228				

*Note:* Unstandardized coefficients (B) are reported with robust standard errors (SE), z statistics, and *p* values based on the Satorra–Bentler scaled test statistic. Std *β* = standardized coefficient; R^2^ = explained variance. T2 = follow‐up.

## 4 Discussion

The present study examined how various risk and protective factors, including baseline depressive symptomatology, perceived burdensomeness, thwarted belongingness, emotion regulation strategies, frequency of NSSI, suicide ideation and meaning in life, were related to future depressive symptomatology in Spanish university students.

Regarding the first objective, the results revealed a positive and significant correlation between baseline and future depression, indicating temporal stability of emotional symptoms over time. This finding suggests that initial levels of depression constitute an important predictor of subsequent distress, reflecting the tendency of these symptoms to remain relatively constant among university populations. In this sense, the study by Roldan‐Espínola et al. (2024) showed that a significant percentage of students maintain depressive symptoms over a 1‐year follow‐up, underscoring the persistence of depressive symptoms during this life stage and the need for early preventive interventions aimed at fostering psychological well‐being in young adults.

In turn, future depression was positively and significantly associated with thwarted belongingness, perceived burdensomeness, and expressive suppression. In this regard, our findings are consistent with those reported by Poindexter et al. (2020), who found that depressive symptoms are closely linked to the interpersonal variables proposed by Joiner's (2005) model among university students, acting as a bridge between adverse interpersonal experiences and increased emotional distress. These results suggest that interpersonal factors may contribute to the maintenance of emotional symptoms by weakening perceived social support and fostering feelings of self‐criticism or social shame, which hinder recovery from depression in university populations (Carrera and Wei 2017).

Concerning the frequency of NSSI, positive and significant relationships were observed with future depression. In this sense, our findings align with those of Whitlock et al. (2013), who found that higher frequency or repetition of NSSI increases the likelihood of maintaining or worsening depressive symptoms among university students. This relationship indicates that repeated engagement in NSSI may function as a maladaptive coping mechanism that, although temporarily relieving distress, contributing to the persistence of depressive symptomatology.

Similarly, suicidal ideation also showed positive and significant correlations with future depression, suggesting that both phenomena could share underlying emotional mechanisms and tend to reinforce each other. This finding is consistent with empirical evidence describing a close and bidirectional relationship between depressive symptomatology and suicidal ideation, with depression being one of the most consistent predictors of the onset and persistence of suicidal thoughts among university students (Luceño‐Moreno et al. 2025).

Furthermore, future depression was negatively and significantly associated with meaning in life, suggesting that this variable may act as a buffer against depressive symptomatology. In line with these data, the meta‐analysis by Wang and Fu (2025), conducted with more than 180,000 university students, showed that meaning in life is positively associated with psychological well‐being and negatively associated with depression and anxiety symptoms, demonstrating a medium protective effect. These findings support the idea that maintaining a clear and stable sense of purpose during university life can foster emotional resilience and reduce the likelihood of experiencing depressive distress over time.

Finally, cognitive reappraisal was also negatively and significantly associated with depressive symptomatology in our study. These results are consistent with previous literature indicating that this emotion regulation strategy promotes better affective and psychological adjustment among university students (Öztekin et al. 2025), as it facilitates a more flexible and adaptive reinterpretation of stressful experiences typical of this stage, reducing their emotional impact and promoting more effective coping strategies when facing academic and personal demands.

Regarding the second objective of the present study, while the correlational analyses showed a significant association between baseline and future depression, the predictive model confirmed that initial levels of depression were the strongest predictor of later depressive symptomatology. In this sense, our findings are consistent with prospective literature indicating that the presence of depression at an initial time point is one of the most robust predictors of recurrence or persistence of future depressive episodes (Fialho et al. 2022). This result reflects a strong temporal stability of emotional symptoms, indicating that prior depressive distress exerts a direct and sustained effect on future distress among university populations, even when other variables in the model—such as thwarted belongingness, perceived burdensomeness, expressive suppression, or meaning in life—are taken into account, as these ceased to show significant effects when baseline depression (T1) was included.

These results are consistent with those of Tagliaferri et al. (2025), who, in a systematic review with young populations, identified previous depressive episodes as the most consistent risk factor for depression recurrence and chronicity, highlighting the importance of early intervention following the first symptoms to prevent the consolidation of emotional distress over time.

Complementarily, our findings relate to the work of X. Liu et al. (2023), which provided specific evidence in university populations and showed that initial levels of depression significantly predict subsequent symptom trajectories. In their 4‐year longitudinal study with university students, the authors identified different depression trajectories characterized by marked temporal stability, such that those with high initial symptom levels were more likely to maintain or intensify their distress over time.

Although baseline depressive symptoms were the strongest predictor of follow‐up depression, the inclusion of interpersonal and emotional variables still offered relevant explanatory value. Specifically, these variables helped identify relational and regulatory processes associated with later depressive symptomatology, even when their direct predictive effects were attenuated after controlling for baseline depression. In particular, thwarted belongingness remained associated with greater expressive suppression and lower meaning in life, suggesting that interpersonal distress may be linked to the maintenance of depressive symptoms through potentially modifiable emotional and existential processes.

This interpretation is consistent with previous research showing that interpersonal difficulties are associated with depressive symptoms in university students (Carrera and Wei 2017; Poindexter et al. 2020), that expressive suppression may contribute to poorer social functioning and reduced social support over time (Srivastava et al. 2009), and that meaning in life is linked to greater psychological well‐being and lower depressive symptomatology (Baquero‐Tomás et al. 2023; Layrón‐Folgado et al. 2022). Therefore, baseline depression may indicate symptom continuity over time, whereas interpersonal and emotional variables may help explain some of the mechanisms through which such distress is maintained, offering clinically relevant targets for prevention and intervention. This interpretation is further supported by the sensitivity analysis excluding baseline depression, in which the role of interpersonal and emotional pathways became more evident, particularly the indirect effect of thwarted belongingness through expressive suppression.

Regarding the third objective of this study, the results indicate that, when initial depression is excluded from the model, future depressive symptoms are primarily influenced by interpersonal and emotional variables. Specifically, thwarted belongingness showed a significant direct and indirect effect on depressive symptoms at T2 through expressive suppression, indicating a partial mediation. In contrast, the indirect pathways involving meaning in life and perceived burdensomeness were not significant. Although thwarted belongingness and perceived burdensomeness were associated with each other and with lower levels of meaning in life, only the pathway linking thwarted belongingness, expressive suppression, and depressive symptoms emerged as a significant predictor. However, perceived burdensomeness also showed a significant direct path to depressive symptoms at T2, indicating a direct prediction but not an indirect effect through the mediating variables included in the model.

These findings suggest that the impact of interpersonal difficulties on depression operates mainly through maladaptive emotional mechanisms, rather than through cognitive or existential processes. In this regard, thwarted belongingness may generate emotional distress that, when managed through expressive suppression, reduces opportunities for receiving social support and emotional validation, thereby intensifying feelings of disconnection and contributing to the maintenance of depressive symptomatology. These results are consistent with those of Davis (2017), who proposed that difficulties in feeling connected to others may increase the risk of depression, especially when individuals rely on maladaptive emotion regulation strategies. Likewise, our findings are consistent with Gupta et al. (2024), who, in a sample of university students, found that greater expressive suppression predicted lower perceived social support, which was linked to higher suicidal ideation—a mechanism that similarly contributes to depressive symptomatology through social disconnection and emotional inhibition.

On the other hand, although thwarted belongingness and perceived burdensomeness were associated with lower meaning in life, this variable did not function as a significant mediator, likely because it represents a more stable and dispositional construct, less sensitive to short‐term fluctuations. Nonetheless, perceived burdensomeness showed a significant direct association with depressive symptoms, suggesting that feelings of being a burden on others may contribute to depressive outcomes independently of emotional or cognitive mediators. In this sample, interpersonal variables—closely related to the experience of unwanted loneliness—appear to play a particularly relevant role in depressive symptomatology, reinforcing the notion that difficulties in establishing satisfying social bonds and feelings of social disconnection are central components in the maintenance of depressive states (Akram et al. 2025; Campbell et al. 2022; Hager et al. 2022). In line with previous research, several authors have suggested that meaning in life constitutes a relatively enduring personal resource linked to psychological well‐being and resilience, rather than an immediate mechanism of change in response to negative interpersonal experiences (King et al. 2006; Steger et al. 2006). Accordingly, its influence may operate more slowly and persistently, while emotional variables—such as expressive suppression—reflect more reactive and maladaptive processes in the face of interpersonal difficulties.

Taken together, the findings highlight that emotional and relational processes act as key mechanisms in the continuity of depression during the university stage. Understanding how these dynamics evolve over time may be crucial for designing preventive interventions that foster adaptive emotional functioning and a stronger sense of belonging.

## 5 Strengths and Limitations

Among the main strengths of the study are its longitudinal design, which allows for the analysis of the evolution of depressive symptomatology based on a broad set of emotional, interpersonal, and existential variables, and the use of regression and mediation models, which contribute to a more accurate understanding of the factors influencing students' psychological distress by identifying both direct effects and underlying mechanisms.

Nonetheless, several limitations should be noted. First, suicidal ideation was assessed using two brief ad hoc items rather than a validated suicide‐specific instrument. This decision was made to reduce participant burden and ensure feasibility within the EMA protocol. Previous research suggests that brief or single‐item indicators of suicidal ideation, including those derived from depression measures (e.g., PHQ‐9 item 9), may provide a valid approximation for screening purposes (Desseilles et al. 2012; Kim et al. 2021). However, this approach may be associated with reduced precision and comparability compared to standardized multi‐item instruments. Therefore, findings involving suicidal ideation should be interpreted with caution. Future studies should incorporate validated suicide‐specific measures to allow for a more comprehensive assessment and to strengthen the psychometric robustness and comparability of results.

Therefore, findings involving suicidal ideation should be interpreted with caution, and future research would benefit from incorporating validated instruments that minimize participant fatigue. Second, the study did not include sex‐disaggregated analyses, which prevented the identification of potential differences in risk and protective patterns between male and female participants. Future research would benefit from including sex as a moderating variable or conducting stratified analyses to explore possible differential pathways.

Finally, the relatively high attrition rate between T1 and T2 represents a limitation of the study and may introduce potential selection bias. Baseline comparisons showed a small but consistent pattern whereby participants who did not complete the follow‐up presented a slightly more vulnerable psychosocial profile, including higher perceived burdensomeness and thwarted belongingness, lower cognitive reappraisal and meaning in life, and a non‐significant tendency toward higher depressive symptoms. However, no differences were observed in key outcomes such as suicidal ideation or non‐suicidal self‐injury, and effect sizes were small, suggesting limited practical impact. Overall, although some degree of selective attrition may be present, its influence on the main findings appears minimal. Nevertheless, the results should be interpreted with caution, as even small baseline differences may have implications over time. High dropout rates are also common in ecological momentary assessment (EMA) studies due to the intensive nature of repeated daily assessments (Burke et al. 2017).

In addition, the 14‐week follow‐up period may have limited the detection of longer‐term changes in depressive symptomatology and related psychological processes. The exclusive use of self‐report measures may also have introduced shared method variance and response biases. Finally, because the sample was drawn from university students recruited in a single geographical and academic context, the generalizability of the findings to other university populations or age groups may be limited.

## 6 Conclusions

The findings of this study indicate that prior depression, together with interpersonal difficulties, NSSI frequency, suicidal ideation, and the use of maladaptive emotion regulation strategies, may constitute a vulnerability pattern characterized by social disconnection and emotional suppression, which, in the university context, increases the likelihood that depressive symptomatology will persist or worsen over time.

Moreover, the results of both structural equation models reveal two complementary patterns. When baseline depression is included, prior symptoms emerge as the most robust predictor of future depression, reflecting a clear temporal stability of emotional distress within the university population. However, when initial depression is removed, the effects of interpersonal and emotional variables emerge more clearly. Specifically, thwarted belongingness showed a significant indirect effect on depressive symptoms through expressive suppression, suggesting that feelings of social disconnection may foster maladaptive emotion regulation patterns that, in turn, maintain or intensify depressive symptomatology.

Taken together, these findings suggest that, beyond the continuity of symptoms, relational and emotional dynamics play a crucial role in the maintenance or exacerbation of psychological distress. This highlights the importance of early interventions aimed not only at reducing depressive symptomatology but also at fostering a sense of meaning in life and enhancing adaptive emotion regulation skills among university students.

## Author Contributions

**José Enrique Layrón:** writing – original draft, conceptualization, investigation, writing – review and editing, supervision. **José H. Marco:** supervision, investigation, methodology. **Rafael Salom Borrás:** supervision, investigation. **Sandra Pérez Rodríguez:** conceptualization, investigation, methodology, formal analysis, supervision, writing – original draft, writing – review and editing, supervision.

## Funding

The authors have nothing to report.

## Ethics Statement

The study was approved by the ethics committee of the Catholic University of Valencia (code UCV2017‐2018‐116). All participants provided informed consent before participating in the study and were free to withdraw at any time without penalty. A safety protocol was implemented for participants reporting elevated suicidal ideation through the app, while confidentiality was preserved through the anonymous design of the study.

## Conflicts of Interest

The authors declare no conflicts of interest.

## Data Availability

The data that support the findings of this study are openly available in Dataverse at https://dataverse.harvard.edu/dataset.xhtml?persistentId=doi:10.7910/DVN/MXXRZK.
